# Effect of peppermint oil and its microemulsion on necrotic enteritis in broiler chickens

**DOI:** 10.14202/vetworld.2021.483-491

**Published:** 2021-02-23

**Authors:** Hend K. Sorour, Reham A. Hosny, Dalia M. A. Elmasry

**Affiliations:** 1 Reference Laboratory for Veterinary Quality Control on Poultry Production, Animal Health Research Institute, Agricultural Research Center, Giza, Egypt; 2 Nanotechnology Research Unit, Animal Health Research Institute, Agricultural Research Center, Giza, Egypt

**Keywords:** broiler chicken, feed conversion ratio, microemulsion, nanotechnology, necrotic enteritis, peppermint oil

## Abstract

**Background and Aim:**

*Clostridium perfringens* is one of the multiple drug-resistant intestinal pathogens causing necrotic enteritis disease, leading to great economic losses in poultry farms. This study aimed to evaluate the potential use of peppermint oil and its microemulsion (ME) as an alternative to antibiotics to control necrotic enteritis in broiler chickens.

**Materials and Methods:**

Peppermint oil ME formulation (15% oil/water) was prepared and characterized by zeta potential, Fourier transform infrared, high-resolution transmission electron microscopy, and liquid chromatography–mass spectrometry (LC-MS/MS). The minimal inhibitory concentrations of the peppermint oil and its ME were investigated. A total of 80 commercial one day old Arbor Acres broiler chickens were randomly assigned to four groups of 20 birds each. The four groups were the negative control, positive control, peppermint oil (0.5 mL/mL water/10 days old), and its ME (0.25 mL/mL water/10 days old) groups. *C. perfringens* was orally provided at concentration of 1×10^8^ CFU/mL on days 14, 15, and 16. Clinical signs and mortality were observed daily. Growth performance, gross lesions and cecal samples were investigated and examined on days 21, 28, and 35.

**Results:**

Peppermint oil ME formulation has a polydispersity index, zeta potential and droplet size of 0.234, −24 mV±4.19, and 29.96±1.56 nm, respectively. LC–MS/MS analysis of oil and ME revealed common presence of phenolic compounds such as rosmorinic (360.31 g/mol), chlorogenic acid (354.31 g/mol), hesperidin (610.56 g/mol), and luteolin 7-O-β-glucuronide (462.1 g/mol). The treated groups with peppermint oil and ME showed lower lesions, mortality and colony-forming units in addition to higher growth performance (p < 0.05) compared to the positive control group.

**Conclusion:**

Our study suggests the potential efficacy of peppermint oil and ME in the reduction of necrotic enteritis lesions and *C. perfringens* count.

## Introduction

Necrotic enteritis is a global disease ­threatening broiler chicken farms. It is characterized by sudden death, decrease in body weight (BW), and diarrhea [1,2]. *Clostridium perfringens* is a causative agent of necrotic enteritis and is considered as a normal flora in the intestinal tract of apparently healthy birds [3-5]. It has different types (A, B, C, D, and E) that are producing various types of toxins (alpha, beta, epsilon, and iota). There are many predisposing catalyzing factors having a role in the proliferation of *C. perfringens* along the intestinal tract, such as dietary components, immune suppression through vaccination, sudden change of feed, coccidial infection, and fasting [6,7]. Misuse of antimicrobial growth promoters in poultry farms for belief of their ability in enhancing growth performance, feed conversion rate, and prevention of the entry of bacterial microbes to poultry flocks has been led to global antimicrobial resistance. Searching for an alternative strategy to antibiotics becomes an urgent need to diminish their use in the poultry sector [8,9].

Natural plant extract materials have been used as a traditional therapeutic remedy in many countries, especially in developing countries [10,11]. Peppermint oil (*Mentha piperita* L.) is extracted from the leaves of the peppermint plant using the steam distillation method [11,12]. Previous reports have displayed the antibacterial activity of the peppermint oil against different bacterial diseases, including *C. perfringens* [12,13]. Plant essential oils (EO) can be used in an emulsion form to increase their antibacterial activity [14,15].

Microemulsions (MEs) are dispersions consisting of an oil phase, water phase, surfactant, and cosurfactant, which are single optically isotropic and thermodynamically stable liquid solutions with a droplet diameter usually within the range of 10-100 nm [16,17]. The advantages of ME over traditional methods are spontaneous formation, thermodynamic stability, the simplicity of manufacture, and high-solubilization capacity for both lipophilic and hydrophilic compounds [18].

The aim of this study was to assess the efficacy of peppermint oil and peppermint oil ME in the control of necrotic enteritis disease in broiler chickens and studying their effect on performance.

## Materials and Methods

### Ethical approval

The experimental study was approved by the Ethical Committee of the Animal Health Research Institute, Ministry of Agriculture, Giza, Egypt.

### Study period and location

The experimental study was conducted in July 2019 for four weeks (days 10 – 35) on 80 commercial 1-day-old Arbor Acres broiler chicken, that were randomly assigned to four groups (20 birds each). This study was conducted in Laboratory Animal Unit, Reference Laboratory for Veterinary Quality Control on Poultry Production (RLQP), Animal Health Research Institute Giza, Egypt. Characterization of peppermint oil ME was done in the Nanotechnology and Advanced Materials Central Laboratory, Agricultural Research center, Giza and in Central laboratory, Faculty of Agriculture, Cairo University.

### Preparation of *C. perfringens* strain

The percentage of necrotic enteritis was 34.7% (8/26), where *C. perfringens* was recorded in eight broiler chicken farms from 26 broiler chicken farms. *C. perfringens* strain (Type A) used in the experimental model was isolated from a field case broiler chicken farm suffering from necrotic enteritis. It was preserved in a brain heart infusion broth containing 25% glycerol at −80°C according to Dar *et al*. [19], Wen and McClane [20]. Detection of the viability and purity of preserved frozen strain was done according to Tessari *et al*. [21] using selective enrichment cooked meat medium (Becton Dickinson and Company, Sparks, Maryland, USA), 10% sheep blood agar with neomycin (40 μg/mL) and tryptose-sulfite-cycloserine agar (TSC) plates, and anaerobic jar containing GasPakTM (Oxoid Limited, Thermo Fisher Scientific Inc., UK) anaerobic incubation. Confirmation of the *C. perfringens* colonies was done as described by Tessari *et al*. [21] using egg yolk agar plates containing 10% Egg Yolk Emulsion (Oxoid Limited, Thermo Fisher Scientific Inc., UK) for detection of the lecithinase activity and litmus milk medium (Oxoid Limited, Thermo Fisher Scientific Inc., UK) for detection of the stormy fermentation reaction.

### Preparation of peppermint oil ME

Peppermint oil was kindly purchased from CAPPHARM^®^, Cairo, Egypt which consists of carvone, menthol and menthone compound (sinod, kadenin, and limonen). Tween 80 was obtained from the Sigma-Aldrich Co.). Double-distilled and deionized water was filtered before use. Peppermint oil ME (oil in water) was prepared in the RLQP by phase titration method according to Rao and McClements [22] using peppermint oil (15 mL), Tween 80 (30 mL), and distilled deionized water (55 mL).

### Characterization of peppermint oil ME

Characterization of peppermint oil ME was done according to Hunter [23], Andrievsky *et al*. [24], and Seung *et al*. [25]. Zetasizer Malvern Instrument (Corp, Malvern, UK) was used to measure droplet size, surface charge (zeta potential), size distribution (polydispersity indexes [PDI]), and electrical conductivity of the ME. Fourier transform infrared (FT/IR)-6100 spectrometer was used to determine the functional groups in the ME based on the bond vibration frequencies among atoms. High-resolution transmission electron microscopy (JEM 1400F HRTEM) was used to analyze the microemulsion shape and size at beam energy of 300 keV.

### Characterization of peppermint oil and peppermint oil ME components using liquid chromatography–mass spectrometry (LC–MS/MS)

#### Chemicals

HPLC grade formic acid was purchased from Merck (Darmstadt, Germany), whereas analytical grade methanol was purchased from Fisher scientific (Leicestershire, UK), Deionized water or HPLC grade water was obtained from Millipore Sigma (Burlington, MA, USA). A 0.45 mm disposable membrane filter was purchased from Cronus Filter (UK).

#### Method

The analysis was carried out in Animal Health Research Institute according to Kapp *et al*. [26] using an Agilent 1260 series (Agilent Co., Santa Clara, CA, USA). Quaternary pump equipped with vacuum degasser, autosampler, thermostatted column compartment, and UV diode array detector interfaced with an Applied Biosystems 4000 QTRAP LC/MS/MS system.

### Determination of antibacterial activity of peppermint oil and peppermint oil ME against *C. perfringens* field strain (*In vitro*)

Antibacterial activity of the two tested chemicals (peppermint oil and peppermint oil ME) against *C. perfringens* Type A strain was assessed as described by Salvia-Trujillo *et al*. [14] and Clinical Microbiology and Infection [27] using the minimum inhibitory concentration (MIC) method. The lowest concentration that showed no bacterial growth had been recorded.

### Anti clostridial effect of peppermint oil and peppermint oil ME in commercial broiler chicken model as a prevention trial

Four weeks experiment was conducted to assess the efficacy of peppermint oil and peppermint oil ME in prevention of necrotic enteritis in broiler chickens.

### Experimental model design

Eighty one-day-old unvaccinated male commercial Arbor Acres broiler chicks were randomly allocated into four groups (20 birds each) in battery cages in Laboratory Animal Unit (biosafety level two) with controlled light and ventilation system in RLQP. Chicks were provided with clean drinking water and weighted diets along the experimental period. A starter diet was administrated during 0 to 13 days, and a grower diet was fed afterward till day 35. The feed met all the nutrient requirements of chicks according to NRC [28].

### Experimental protocol

Experimental protocol was according to Eid *et al*. [29], with some modifications in the induced stress factor; before starting of the experiment, 10 chicks (extra than 80 chicks) and feed were exposed to bacterial investigation for (*E. coli*, *Salmonella*, and *C. perfringens*) using MacConkey, XLD, and sheep blood agar plates, respectively, and confirmed using different biochemical tests according to ISO6579-1 2017 [30], Dufour-Zavala [31], and Lovland *et al*. [32]. Chicks were assigned to four groups as group 1: Negative control, group 2: Positive control (*C. perfringens*), group 3: Peppermint oil (0.5 mL/mL water), and group 4: ME (0.25 mL/mL water) groups. All chicks in all groups were exposed to 12 h feed withdrawal time on day 13. Group I was administrated with 1ml of Brain heart infusion broth on days 14, 15, and 16. Groups (two, three, and four) were challenged with 1 mL (1×10^8^) *C. perfringens* as three successive doses for days 14, 15, and 16. Groups (three and four) were treated with peppermint oil and peppermint oil ME, respectively, through drinking water on day 10^th^ till the end of the experimental period 35^th^ day. The dose of peppermint oil and ME used in groups (three and four) was calculated according to results of the MIC.

### Laboratory examination

Clinical signs and mortality were observed daily during the experiment. On days 21, 28, and 35 of the experiment, samples were taken from the intestine and liver for the examination of gross lesions as described by Tizhe *et al*. [33]. Furthermore, cecal samples were examined for *C. perfringens* counts using Reinforced clostridial agar plates as described by Brady [34]. Performance parameters was assessed according to Ensminger [35], and Barzegar *et al*. [36] through measurement of body weight (BW), body weight gain and feed conversion ratio (FCR) for each group during the experiment.

### Statistical analysis

Statistical analysis was conducted using SPSS IBM 21 software, (IBM Corp., Armonk, NY) to evaluate the reduction of *C. perfringens* counts throughout the experimental groups using non-parametric tests (Kruskal–Wallis and Mann–Whitney tests). *C. perfringens* counts did not show a normal distribution according to Kolmogorov–Smirnov test (n>50).

Furthermore, the differences in BW gain and FCR in different groups were analyzed using (one-way analysis of variance). Results of all tests used in this study were considered significant at p≤0.05.

## Results

### Preparation of *C. perfringens* strain

The colonies of *C. perfringens* were identified as double zone of hemolysis on sheep blood agar with neomycin medium and black colonies on TSC medium. Furthermore, suspected colonies were confirmed using biochemical tests and revealed positive lecithinase activity on egg yolk salt agar, positive stormy fermentation on litmus milk medium.

### Determination of antibacterial activity of peppermint oil and peppermint oil ME against *C. perfringens* field strain (*In vitro*)

The efficacy of peppermint oil and peppermint oil ME against *C. perfringens* was evaluated using minimal inhibitory concentration method. The results revealed that the MICs of peppermint oil and peppermint oil ME were 0.5 mL/L and 0.25 mL/L, respectively.

### Characterization of peppermint oil ME

The peppermint oil ME (15% oil/water) was characterized for the conductivity, viscosity, PDI, and zeta potential that revealed 0.0151 ms/cm, 0.8872, 0.234, and −24 mV ±4.19, respectively.

**Figure-1:**
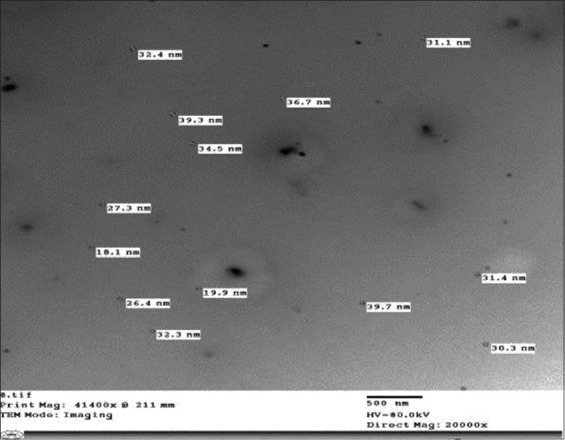
High-resolution transmission electron microscopy of peppermint microemulsion revealed that droplets size 29.96 nm±1.56, there are no aggregation, size homogeneity, and spherical nature.

**Figure-2:**
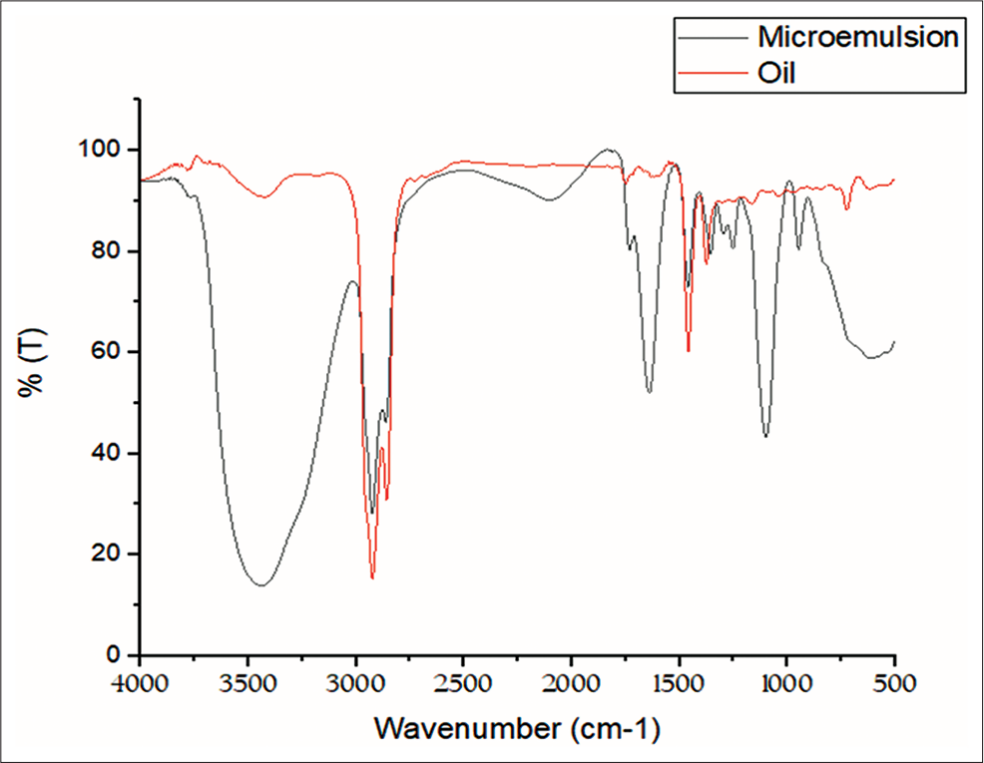
Fourier-transform infrared spectroscopy of peppermint oil and peppermint microemulsion.

The results of HRTEM (Figure-1) revealed that peppermint oil ME had no aggregation, size homogeneity, and spherical nature. The average size of peppermint oil ME was 29.96 nm ±1.56 The distinguish of FT-IR bands in ME versus peppermint oil was divided into two regions: 1) The fingerprint region in ME had vibration bands of <1500 cm^−1^ :1295.93 cm^−1^ and 1251.58 cm^−1^ for Ester carbonyl, phenol and phenyl, acryl C-O, respectively. 1251.58 cm^−1^ for strong C-O stretching of aromatic ester group, 945 cm^−1^ and 913 cm^−1^ for strong O-H bonding of carboxylic acids group 2) The group frequency region in ME had vibration band of 2102.99 cm^−1^ that assigned to weak C Ξ C stretching of alkyne group. The FT-IR analysis of peppermint oil and ME exhibited same band pattern with different intensities below 1500 cm^−1^: (1458.29 cm^−1^, 1459.85 cm^−1^) for C-H bonding alkane of methylene group, (1035 and 1098.26 cm^−1^) for C–O–C bonding of secondary alcohol, and (1374.04 and 1354.75 cm^−1^) for phenol – alcohol groups, respectively. Peppermint oil and ME exhibited same band pattern with the same intensities in the group frequency region at a vibration band of 2923.56 that assigned to CH3 of alkanes. Furthermore, peppermint oil and ME exhibited same band pattern with different intensities in the group frequency region (3424 and 3438 cm^−^1) for H bonding and O-H group of phenol and alcohol group, (2857.99 and 2861.82 c^m−^1) for C-H stretching of alkanes, (1748 and 1730 c^m−^1) for C=O stretching of carbonyl group, and (1630 and 1639.2 c^m−^1) for N-H stretching of primary amines, respectively. (Table-1 and Figure-2). LC–MS/MS analysis of oil and ME revealed common presence of some phenolic compounds such as rosmarinic (360.31 g/mol) and chlorogenic acid (354.31 g/mol), hesperidin (610.56 g/mol) and luteolin 7-O-β-glucuronide (462.1 g/mol) and non-phenolic compounds such as menthone (154.25 g/mol)., Tween 80 (1309.68 g/mol) was only observed in the ME whereas Carvone (150.22 g/mol) was only observed in peppermint oil.

**Table-1:** Fourier transform infrared spectroscopy described fingerprint bands of peppermint oil and microemulsion.

Microemulsion bands	Peppermint oil bands	Functional groups
-	3781.72	(–OH stretching)
3438	3424	(–OH stretching)
2923.56	2923.56	CH3 groups
2861.82	2857.99	CH2 groups
2102.99	-	Weak C C stretching alkyne group
1730	1748	C O bonding
1639.2	1630	N H stretching primary amines
1459.85	1458.29	C H bonding methylene group
1354.75	1374.04	Phenol and alcohol group
1295.93	-	Ester carbonyl group and phenol
1251.58	-	Phenyl and acryl C O
1098.26	1035	C–O–C bonding
945	-	O H bond carboxylic acids
913	-	O H bond carboxylic acids

### Laboratory examination of the experimental model

No signs were observed during the experimental period in negative control, peppermint oil and peppermint oil ME groups (one, three, and four). signs were only detected in the positive control group (two) within 4 to 21 days post-challenge including diarrhea, reduction in body weight, emaciation and reluctance of movement (Table-2).

**Table-2:** Clinical signs and postmortem examination in *Clostridium perfringens* challenged groups.

	Number of examined birds	Mortality rate	Symptoms						Gross lesions					
	
			Diarrhea	Unable movement	Sudden death	Reduction in body gain	Emaciation	Intestinal lesion (along small intestine duodenum jejunum ileum) also lesion include cecum					Liver lesion	
	
								Distention with gases	Enteritis	Yellow necrotic membrane	Unabsorbed and undigested feed	Hemorrhage	Congestion	Multiple yellow necrotic foci
21 day (7 days P.I)														
G1−ve	20	2/20	N	N	D	NBW	N	N	N	N	N	N	N	N
G2+ve	20	7/20	Slight	One	D	RBW	N	D	D	N	N	N	N	N
G3 Oil	20	1/20	N	N	D	NBW	N	N	N	N	N	N	N	N
G4 microemulsion	20	2/20	N	N	D	NBW	N	N	N	N	N	N	N	N
28 day (14 days P.I)														
G1−ve	13	0/13	N	N	0%	NBW	N	N	N	N	N	N	N	N
G2+ve	8	4/8	Diarrhea	One	D	RBW	D	D	D	D	D	D	D	D
G3 oil	14	1/14	N	N	D	NBW	N	2/5	2/5	N	N	N	N	N
G4 microemulsion	12	0/13	N	N	D	NBW	N	2/5	2/5	N	N	N	N	N
35 day(28 days P.I)														
G1−ve	8	3/8	N	N	D	NBW	N	N	N	N	N	N	N	N
G2+ve	2	0/2	Diarrhea	One	0%	RBW	D	D	D	D	D	D	D	D
G3 oil	9	0/9	N	N	0%	NBW	N	0/5	0/5	N	N	N	N	N
G4 microemulsion	8	0/8	N	N	0%	NBW	N	0/5	0/5	N	N	N	N	N

D=Detected, N=Not detected, NBW=Normal body weight, RBW=Reduction body weight, P.I.= Post infection

No gross changes were observed in peppermint oil and peppermint oil ME groups (groups three and four) in intestine and liver except distention of intestine with gases and enteritis on day 28 day. Gross lesions were observed in the positive control group (two) such as intestinal distension with gases, enteritis at 7-day post-challenge and increased at 14- and 21-day post-challenge including enteritis, scattered necrotic foci along the mucosa, presence of yellow diphtheritic necrotic membrane, gaseous distention, hepatic congestion, multiple yellow necrotic foci in liver and presence of undigested feed (Table-2). Dead birds in the positive control group (group two) during the experimental period showed severe degree of gross lesions.

**Figure-3:**
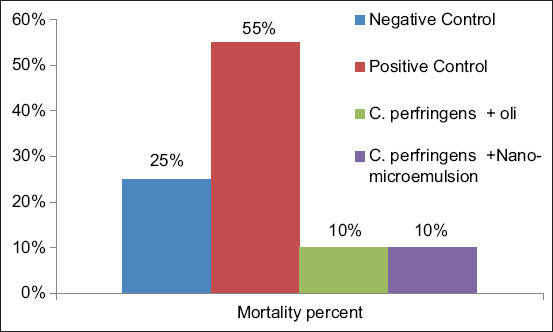
Mortality rate in broiler chickens during the experimental treatments.

Treatment with peppermint oil and peppermint oil ME was reduced the mortality rate in the groups three and four to 10% (2/20) compared to the positive control group (II) that showed a high mortality rate of 55% (11/20) (Figure-3).

**Figure-4:**
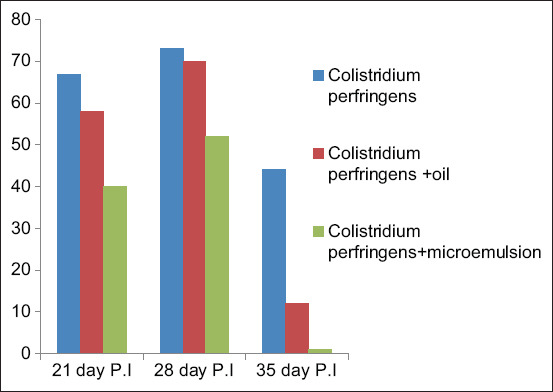
Quantitative determination of cecal *Clostridium perfringens* counts using Reinforced Clostridial agar. P.I.=Post infection.

The peppermint oil and ME were most effective on day 35 of the experimental period resulting in a significant reduction of cecal *C. perfringens* counts in groups three and four by Kruskal – Wallis test (p<0.05) compared to the positive control group (group two) (Figure 4). There was no significant difference among the administration of peppermint and peppermint oil ME treatments by the Mann-Whitney test (p<0.05).

Treatments with peppermint oil and peppermint oil ME in groups three and four were significantly increased the BW gain, improve total feed intake and decrease FCR using one-way ANOVA test (p<0.05) compared to the positive control group (group two) (Tables-3 and 4).

**Table-3:** Body weight and feed intake of broiler chickens during the *Clostridium perfringens* challenged experiment.

Groups	Body weight (g)					Feed intake/bird	
	
	Zero day	14 days	21 days	28 days	35 days	21 days	35 days
Group 1 (−ve)	47.69±0.89	375.18±6.03^a^	804.1±8.06^a^	1357.09±11.54^a^	1937.26±19.27^a^	1380.66±0.38^b^	3303.28±0.32^a^
Group 2 (+ve)	46.68±0.74	327.3±7.70^b^	724.23±16.87^b^	1312.36±12.31^bc^	1822.64±18.12^b^	1301.91±0.55^b^	3148.24±0.31^b^
Group 3 (peppermint oil)	47.43±0.80	349.12±8.23^ab^	767.27±15.52^b^	1321.54±20.75^b^	1835.63±33.68^b^	1246.48±0.25^a^	3114.31±0.04^c^
Group 4 microemulsion)	46.48±0.72	363.87±7. 68^a^	793.15±17.23^ab^	1345.43±13.01^a^	1889.96±32.15^ab^	1313.42±0.17^b^	3219.1±0.09^ab^

N.B. The different letters of columns denote significant variations between means (p≤0.05)

**Table-4:** Body weight gain and feed conversion rate of broiler chickens during the experimental treatments.

Group	Body weight gain		Feed conversion rate	
	
	21 days	35 days	21 days	35 days
Group 1 (−ve)	756.41±17.49^a^	1889.57±43.25^c^	1.83±0.012^b^	1.75±0.02^a^
Group 2 (+ve)	677.55±12.07^c^	1775.96±59.3^a^	1.92±0.03^a^	1.77±0.014^a^
Group 3 (peppermint oil)	719.84±19.61^b^	1788.2±21.28^a^	1.73±0.04^a^	1.74±0.03^a^
Group 4 (microemulsion)	746.67±6.73^a^	1843.48±26.63^b^	1.76±0.012^b^	1.75±0.018^a^

N.B. The different letters of columns denote significant variations between means (p≤0.05)

## Discussion

The characterization of peppermint oil and ME is a first step towards determining the efficacy of the peppermint oil and ME in the prevention of necrotic enteritis. Our peppermint oil ME formulation (15% oil/water) have PDI, zeta potential, and droplet size of0.234, −24 mV ±4.19 and 29.96±1.56 nm, respectively that agree with Lv *et al*. [37] who reported similar observations regarding the lower droplet size and PDI of peppermint oil ME and this attributed to the type of surfactant used; tween 80 (70 nm) has a significant influence on droplet size and polydispersity index.

Marcela *et al* [38] has displayed that peppermint oil ME formulation (30% oil/water) had particle size, conductivity, viscosity, PDI, and zeta potential of 12.66±0.54 nm, 60.0±3.3 μS/cm, 3.58, 0.265, and −4.35 mV ±4.19, respectively. The infrared spectra of peppermint oil and ME after infusion mainly exhibited same band pattern representing phenolic and non-phenolic compounds indicating that no noticeable changes occurred during the formulation.

The anticlostridal activity of peppermint oil and ME observed in this study agrees with a previous report of Encun *et al*. [39] who displayed the antimicrobial activity of peppermint EO against *C. perfringens in vitro* and *in vivo* . Our study shows that a high BW gain was observed in both treated groups 3 and 4. Similarly some EO products revealed antimicrobial activity against *C. perfringens* and had the ability to elevate productive performance in poultry [40].

The severity of signs, gross lesions and mortalities observed in the challenged chickens with *C. perfringens* (1×10^8^) in the positive control group (group 2) may be attributed to the dietary stress factor induced through the application of feed withdrawal period of 12 hours on day 13. These findings agree with a previous report of Redondo *et al*. [5] who displayed the application of feed withdrawal in birds challenged with *C. perfringens* type A strain develops severe gross lesions in 70% of challenged birds.

On the other hand, a study by Olkowski *et al*. [41] has displayed the subclinical form of necrotic enteritis in the challenged chickens with *C. perfringens* Furthermore, Our study displayed the reduction of the severity of signs lesions and mortalities in both treated groups with ME and peppermint oil, this agrees with results reported by Timbermont *et al*. [42] and Jerzsele *et al*. [43] who displayed the addition of EO can lead to a decrease in morbidity percentage in birds developing necrotic lesions in the gut, increase BW gain, and diminish postmortem lesions compared to the control group.

A study by Ameri *et al*. [44] has displayed that broilers whose diets were supplemented with peppermint powder had higher BW on day 42 . Feed supplementation with a blend of peppermint and eucalyptus EO improved the immune status and performance of broiler chickens infected with the Newcastle disease virus, and increased their final BW, decreased final FCR, and total mortality [45]. The performance of broilers whose diets were supplemented with different doses of dry peppermint was observed with a higher weekly WG and lower FCR in chicks that were fed with a lower (0.5%) than a higher (1.5%) peppermint dose [46]. The increase BW gain and lower FCR observed in this study in broiler chickens fed the peppermint diet in groups three and four may be related to the digestibility and gut microbial balancer characteristics of menthol that stimulate the secretion of endogenous digestive enzymes, and improving poultry growth [47].

Diets containing peppermint leaves were reported to improve the growth performance of broiler chicks at early stages of life [48].

Furthermore, the supplementation of dried *Mentha pulegium* at 5 g/kg improved growth performance of broilers on day 42 [49]. BW, BW gain, and FCR were significantly increased (p<0.05) for broilers fed on 1.5% peppermint leaves compared to the control group [50]. On the other hand, a reported case [51] has revealed that the supplementation of dried *Mentha cordifolia* at concentrations of 0.5%, 1.0%, 1.5%, and 2.0% had no significant effect on the growth performance of broilers on day 42. The results of this study support the observations of Spirling and Daniels [52] who reported that peppermint has a positive effect on digestion and can strongly improve feed intake.

Our study has displayed the reduction of cecal *C. perfringens* count in both treated groups with peppermint oil ME and peppermint oil compared to the positive control group which agrees with previous reports [53-56] that displayed the use of some EO decreased *C. ­perfringens* counts in poultry.

## Conclusion

Our study suggests the potential use of peppermint oil and ME in the reduction of necrotic enteritis lesions and *C. perfringens* count.

## Authors’ Contributions

HKS and RAH performed all bacteriological work. DMAE performed preparation and characterization of microemulsion, designed and coordinated the study. HKS, RAH, and DMAE designed the work and performed the experiment. RAH and DMAE performed all statistical analysis and wrote the manuscript. All authors read and approved the final manuscript.
